# Physical activity as a risk factor for amyotrophic lateral sclerosis-findings from three large European cohorts

**DOI:** 10.1007/s00415-020-09995-x

**Published:** 2020-06-23

**Authors:** Hu M, Robertson NP

**Affiliations:** 1 Department of Neurology, Royal Gwent Hospital, Cardiff Road, Newport, Gwent NP20 2UB Wales, UK; 2 Department of Neurology, Division of Psychological Medicine and Clinical Neuroscience, Cardiff University, University Hospital of Wales, Heath Park, Cardiff, CF14 4XN UK

## Introduction

There has been a longstanding historic interest in a possible link between amyotrophic lateral sclerosis (ALS) and levels of physical activity, fuelled by high profile athletes intermittently acquiring the disease and raising its profile. Putative mechanisms behind this hypothesis remain unclear but have implicated; oxidative stress, glutamate excitotoxicity, glial activation, anti-apoptotic proteins, neurotrophic factors, and exercise-induced changes in neuron morphology. Previous small observational studies of athletes have suggested certain groups of athletes (including soccer or American football players) may have an increased risk of ALS. More recently, larger scale studies have explored the association of ALS with physical activity in general. The methodology underpinning these studies has been mixed, and the strengths and directions of association have thus far been inconsistent.

In this month’s journal club we discuss two recent papers examining links between current and historical physical activity and ALS incidence and mortality in general populations. We also examine a large-scale paper comparing how ALS risk might potentially vary with level of athletic attainment in a selective group of skiers.

## Multicentre, cross-cultural, population-based, case–control study of physical activity as risk factor for amyotrophic lateral sclerosis

In this large multicentre retrospective population-based case–control study, the authors examined historical levels of physical activity and the risk of developing ALS. The data were gathered from 5 centres in 3 European countries (Netherlands, Irelands and Italy), and was based on analysis of data from 1557 ALS patients and 2922 matched controls.

A comprehensive written structured questionnaire was used by all five centres to collect demographic and lifestyle data, and to estimate total historical physical activity. Participants were asked to recall all occupation and work-related activities throughout their life. In addition they were asked to describe and quantify all historical recreational sport and leisure activities. All activities were scored and coded using the Compendium of Physical Activities and ascribed a Metabolic Equivalent of Task (MET) ratio. The authors also attempted to adjust for other potential confounding factors, such as age, gender, smoking, alcohol intake and toxin exposures.

There was a positive linear association between the history of physical activity, in leisure time (OR [odds ratio] 1.07, *P* = 0.01), occupational activities (OR 1.06, *P* < 0.0005) and all activities (OR 1.06, 95% CI [confidence interval] 1.04–1.09, *P* < 0.001) and risk for the development of ALS. The increase in ALS risk associated with physical activity was independent of confounders such as age, gender, education, smoking and alcohol. Adjustment for past history of head injury did not alter the odds ratios.

The authors suggest that the more active a person has been over their lifetime, the greater their risk of developing ALS. Although this was most apparent in those who had been very vigorously active, even moderate levels of activity (such as leisure activities) were associated with an increased ALS risk.

### Comments

This is a large, well-constructed multi-centre trial utilising a range of clinical and demographic data. Strengths include the use of lifetime (rather than just current) physical activity estimated using standardised methodology (in contrast to some previous studies), the care taken to control for possible confounding risk factors and utilisation of genetic information (separately analysing those with C9orf72 mutation). Weaknesses include the possibility of interview bias in data collection, with face to face interviews not performed in the Netherlands (the biggest recruitment area), where the strength of association was weakest. In addition, although note is made of site of onset of disease in demographics, this is not included in the analysis. Furthermore, homemaking duties and military service (during which physical activity is likely to be high) are excluded from the analysis and using self-reported lifetime physical activity in a group of ALS patients (who likely have impaired physical activity) may also increase risk of recall bias.

Visser A et al (2018) J Neurol Neurosurg Psychiatry 89:797–803

## Physical activity and risk of amyotrophic lateral sclerosis in a prospective cohort study

In this large prospective cohort study, the authors set out to determine if there was a relationship between current physical activity and the subsequent risk of dying from ALS. 472,100 participants aged between 35–70 years were included in the analysis. They were recruited from 23 centres across 10 Western European countries between 1992 and 2002, and followed up for several years.

Total baseline physical activity for the last year was investigated using the Cambridge Physical Activity Index (CPAI), incorporating comprehensive measures of physical activity across a wide array of occupational and recreational tasks. ALS cases were defined as those in whom ‘motor neuron disease’ was subsequently reported as an immediate, antecedent or underlying cause of death.

A total of 219 ALS deaths (76 men and 143 women) arose during follow-up, with the mean follow-up period being 13 years (SD 3 years). Total physical activity was inversely associated with ALS mortality, with a significant trend across categories (*P* = 0.042) in the fully adjusted model. Those who were physically active were 33% less likely to die from ALS compared to those who were inactive, with a hazard ratio (HR) of 0.67 (95% CI 0.42–1.06). Although factors such as age, gender, BMI, education and smoking did not confound the results, introducing waist-hip-ratio lowered the significance of the trend (*P* = 0.084).

This is the first prospective cohort study on ALS and physical activity, in which the authors conclude that there is a slightly decreased risk of dying from ALS in those with high levels of physical activity at enrolment. This does not seem to be confounded by age, gender, anthropometry, smoking and education.

### Comments

This is a large study which followed up 472,100 subjects across 5,815,773 person-years. The prospective nature of the study allows elimination of some of the recall bias inherent in retrospective studies and the authors also attempted to control for a number of potential confounding factors. To minimise the potential influence of reverse causality the authors excluded ALS deaths occurring in the first 3 years of follow- up.

Of note the cohort seems to over-represent females (68.6% of the total participants) for reasons that are unclear and the analysis utilises ALS-related deaths as the outcome, thus potentially underestimating the number of total ALS cases in the cohort. The authors measured physical activity only for the year preceding enrolment, meaning we have no information for the levels of physical activity in the intervening years prior to ALS related death, or indeed in earlier life. Additionally, we do not know if site of disease onset, disease severity or duration of illness may be influenced by physical activity.

Gallo V et al (2016) Eur J Epidemiol 31:255–266

## Amyotrophic lateral sclerosis among cross-country skiers in Sweden

In this large retrospective cohort study in Sweden, researchers studied the risk of developing ALS in a cohort of cross-country skiers and a control group of non-skiers. 212,246 professional and amateur skiers who had participated in the 90 km Vasaloppet long distance race were compared against 508,176 general Swedes between 1989 and 2010. Furthermore, the skiers were further divided according to their race performance. Subsequent incidence of ALS was gathered using individual linkages of participants with the Swedish Patient Register and Causes of Death Register.

39 ALS cases were identified in the skiers (incidence rate 2.57 per 100,000). Skiers with the fastest finishing times (100–150% of winner time) had a 4.31-fold risk of ALS (95% CI 1.78–10.4) and those with 151–180% of winner time had 2.70-fold risk of ALS (95% CI 1.14–6.35) when compared to the slowest finishers (> 180% winner’s times). Those who had the fastest times and participated in more than 4 races appeared to have a > 12-fold risk of ALS (HR 12.2, 95% CI 2.30–64.6) compared to the slowest finishers who only participated in one race.

A total of 150 ALS cases were identified among the non-skiers. Compared to the non-skiers, the fastest skiers (HR 2.08, 95% CI 1.12–3.84) and skiers with > 4 races (HR 1.88, 95% CI 1.05–3.35) both had a higher risk of ALS, whereas the slower skiers (> 180% of winner time) had a decreased risk of ALS (HR 0.46, 95% CI 0.24–0.87) compared to the non-skiers.

The authors suggest that long distance cross-country skiing is associated with a higher risk of ALS, but only among the best skiers. They also suggest that recreational skiers appear to have a reduced risk when compared with non-skiers.

### Comments

This is a very large cohort of athletes and controls. The authors made use of the comprehensive nationwide Swedish Patient Register, and had access to demographic information such as education, employment and region of residence. Utilising race performance data allowed the authors to perform internal comparison between the skiers, as well as external comparison between skiers and non-skiers. Cross-country skiing itself is a very demanding endurance sport but with low levels of head injury and trauma, potentially removing the CTE hypothesis as a confounder.

Despite the large numbers at recruitment, the relative few numbers of ALS cases in the skiers (39) does make extrapolation difficult. There are also inherent difficulties with the accuracy of utilising large-scale registers as the primary source of ALS diagnosis, with potentially inaccurate coding or missing ALS cases. In this cohort there may be other potential confounding exposures, such as the use of performance enhancing drugs in the professional athletes. The cohort of Swedish cross-country skiers is a highly selective one, making the results difficult to generalise across other sports or general populations.

Fang F et al (2016) Eur J Epidemiol 31:247–253

## Discussion

The three studies show interesting, but divergent evidence about the links between physical activity and ALS. Visser et al. suggest that historical physical activity increases the risk of developing ALS, whilst Gallo et al. suggest that high levels of current physical activity might reduce the risk of dying from ALS. Fang et al. meanwhile suggest that whilst elite athletes might have an increased risk of ALS and recreational athletes might have a reduced risk when compared to the general population.

These studies highlight the difficulties in studying the factors influencing a rare disease such as ALS at a population level. It is important to note that the studies report association, and we cannot infer causality from the data presented. We still have no clear evidence that it is physical activity itself that influences the incidence of ALS, rather than the demographic, lifestyle and genetic factors associated with those who exercise more. We also do not know enough about the early (pre-symptomatic) stages of ALS from a cellular and biological level, and how that might influence physical activity levels. Although many pathophysiological mechanisms linking ALS and physical activity sound plausible, they remain speculative.

Much of the current research has relied on historical or retrospective data, future prospective studies may also help us elucidate the strengths and directions of relationship more clearly. Further studies might also make use of other patient populations, rather than focussing on Western European cohorts. In addition studies exploring physical activity and genetics might allow testing of gene-environment interactions and modern technologies, including fitness trackers and smart apps may provide more accurate data on physical activity levels.

The links between physical activity and ALS have already led to some alarming newspaper headlines. We must bear in mind that physical activity has been demonstrated to be protective against many diseases including cardiovascular disease, diabetes and a variety of cancers. The positive effect of physical activity on these common conditions may prove more important than the risk of a serious but far less common disease like ALS.

